# Differential Binding of Tetrel-Bonding Bipodal Receptors to Monatomic and Polyatomic Anions

**DOI:** 10.3390/molecules24020227

**Published:** 2019-01-09

**Authors:** Steve Scheiner

**Affiliations:** Department of Chemistry and Biochemistry, Utah State University, Logan, UT 84322-0300, USA; steve.scheiner@usu.edu; Tel.: +435-797-7419

**Keywords:** Sn, bidentate, formate, HSO_4_^−^, H_2_PO_4_^−^

## Abstract

Previous work has demonstrated that a bidentate receptor containing a pair of Sn atoms can engage in very strong interactions with halide ions via tetrel bonds. The question that is addressed here concerns the possibility that a receptor of this type might be designed that would preferentially bind a polyatomic over a monatomic anion since the former might better span the distance between the two Sn atoms. The binding of Cl^−^ was thus compared to that of HCOO^−^, HSO_4_^−^, and H_2_PO_4_^−^ with a wide variety of bidentate receptors. A pair of SnFH_2_ groups, as strong tetrel-binding agents, were first added to a phenyl ring in ortho, meta, and para arrangements. These same groups were also added in 1,3 and 1,4 positions of an aliphatic cyclohexyl ring. The tetrel-bonding groups were placed at the termini of (-C≡C-)_n_ (*n* = 1,2) extending arms so as to further separate the two Sn atoms. Finally, the Sn atoms were incorporated directly into an eight-membered ring, rather than as appendages. The ordering of the binding energetics follows the HCO_2_^−^ > Cl^−^ > H_2_PO_4_^−^ > HSO_4_^−^ general pattern, with some variations in selected systems. The tetrel bonding is strong enough that in most cases, it engenders internal deformations within the receptors that allow them to engage in bidentate bonding, even for the monatomic chloride, which mutes any effects of a long Sn···Sn distance within the receptor.

## 1. Introduction

The extraction and transport of anions are of great importance in a wide diversity of applications [1]. Their detection, even in minute quantities, is no less important. But it is one thing to detect the presence of a generic anion, and quite another to do this in a highly selective fashion. For example, it may be necessary to bind, transport, or extract a particular anion in a given process, over and above another halide. It is for this reason that evolution has developed a panoply of anion-binding proteins. The sulphate-binding protein of *Salmonella typhimurium* [2] is an example of one which binds this anion via a number of H-bonds. Another protein is responsible for the binding and transport of phosphate [3] with very high specificity, and yet another protein is highly specific for the nitrate anion [4], while another binds specifically to bicarbonate [5]. Whereas biological evolution has developed some very specific and selective anion binding agents, modern technology lags far behind. Many receptors make use of general electrostatic interactions, and sometimes employ H-bonds [6,7,8,9]. However, the anion receptors that have been developed to date still suffer from certain disadvantages. Their selectivity lags behind what is needed, or they are unable to detect the presence of a particular anion below a given concentration threshold.

One major, and fairly recent, advance in this field has arisen with the growing recognition of the phenomenon of halogen bonds (XBs) [10,11,12,13,14,15,16,17]. These XBs have been thoroughly dissected by theoretical calculations over the past years, and are now quite well understood. One of the more intriguing and potentially useful applications of XBs is associated with the development of receptors that are highly selective for one anion over another [18,19,20,21,22,23,24]. In an early effort in this direction, the Beer group [25] found that the substitution of H by Br enabled the consequent XB to more effectively bind chloride. They later showed [26] that receptors of this type could recognize both chloride and bromide ions, purely by virtue of XBs, and demonstrated an increased affinity over H-bonding analogues [27,28]. Work since that time has led to further advances in the application of XBs as important elements in anion receptors [29,30,31,32]. Quantum calculations have added further insights into some of the important principles involved [33,34,35].

Just as the switchover from H to halogen bonding introduced a new dimension to the field, extending this same philosophy to another sort of bonding offers even more important potential benefits. More specifically, just as the elements of the halogen family (Cl, Br, I, etc) can replace H as a bridging atom in strongly bound complexes, the same is equally true for other families in the periodic table. In particular, chalcogen [36,37,38,39,40,41,42], pnicogen [43,44,45,46,47,48,49,50,51], and even tetrel atoms [52,53,54,55,56,57,58,59,60] have been shown to engage in noncovalent bonds that share the name of the element family. Importantly, this sort of bonding can occur in the context of anion receptors, as shown by some very recent work [61,62,63,64,65,66,67,68] wherein it is a set of chalcogen bonds that enable a newly synthesized set of molecules to bind to and transport anions. Quantum calculations have entered the fray and shown that the transition from chalcogen to pnicogen to tetrel yielded [69,70] not only progressively stronger binding to anions, but also improved selectivity.

Previous work has identified tetrel bonds as offering the optimal combination of strong binding along with a high degree of selectivity in a competition between various anions [69,70,71,72]. Amongst these tetrel atoms, it is Sn that is the best in these two categories. In fact, the excellent ability of Sn to bind ions has been extensively explored by Jurkschat et al. [73,74,75] in a number of experimental papers. Therefore, it is to the tetrel bonds formed by Sn atoms that the current work is devoted. The second issue has to do with the particular anions. Earlier work focused on halides as anions to be differentiated by a receptor. But of course, it is not just simple monatomic halides that are important in this field, but also larger polyatomic species. As such, it is entirely possible, even probable, that a larger anion might be better able to span the distance between the two Sn atoms in a bipodal receptor. One can imagine a receptor designed with an Sn··Sn distance and orientation that perfectly enables the two O atoms of an anion such as HCOO^−^ to fit snugly, with each O atom interacting with one of the two Sn atoms in a strong and highly directional tetrel bond. The inter-tin distance might be too long for a monatomic halide to engage in more than one Sn··X tetrel bond, leaving it at a disadvantage. On a more refined level, the HCOO^−^ anion ought to have somewhat different geometrical binding criteria than a nominally similar H_2_PO_4_^−^, as well as different electronic characteristics that affect their tetrel binding.

The current work represents an inquiry into these issues. Can one design a bipodal anion receptor, one that binds via Sn-tetrel bonds, that is optimized for one particular anion over others? For this purpose, HCOO^−^, HSO_4_^−^, and H_2_PO_4_^−^ were taken as representative polyatomic monoanions. What they have in common is that they can all bind to the Sn through two O different atoms. However, each anion has a different set of inter-oxygen distances and pertinent angular characteristics that are important in tetrel bonding. As a point of comparison, Cl^−^ was taken as a model halide. It engages in strong tetrel bonds with Sn, but not so strong as to be considered a covalent bond, as is the case [71,72,76] with F^−^. But importantly, as a monatomic, it would have difficulty in spanning the entire distance between Sn atoms, so offers a strong point of contrast in this regard.

Bipodal receptors take, as their initial model, a phenyl ring on which two -SnFH_2_ groups are placed. The intense σ-hole that lies opposite the F atom is ideally situated to form a strong tetrel bond. These two groups are placed ortho, meta, and para to one another, generating a wide range of bonding possibilities. The effects of aromaticity of the receptor ring are examined by replacing phenyl with an aliphatic cyclohexyl ring. As a means of placing the two SnFH_2_ groups further apart, they are situated on one or more -C≡C- spacer groups, which are again either ortho or meta to one another on the base phenyl ring. As a last issue, instead of placing the SnFH_2_ groups as substituents on these rings, they are directly incorporated into the ring structure in an aliphatic eight-membered ring.

## 2. Computational Methods

The Gaussian-09 [77] program (Gaussian Inc., Wallingford, CT, USA) was employed for all calculations which were carried out with the M06-2X DFT functional, which has shown itself to be reliable and accurate for related systems [78,79,80,81,82,83,84]. The aug-cc-pVDZ basis set was used for all atoms, with the exception of Sn, for which the aug-cc-pVDZ-PP pseudopotential from the EMSL library [85,86] was applied so as to account for relativistic effects. All geometries were fully optimized, and checked to ensure they were true minima, containing all positive vibrational frequencies. The binding energy, E_b_, of each complex was defined as the difference between the energy of the complex and the sum of the energies of separately optimized monomers. Interaction energies were similar, but refer to the monomers in the geometries they adopt within the complex. Basis set superposition error was removed via the counterpoise [87,88] procedure. Free energies at 298 K were computed using standard physical chemistry formulae. Molecular electrostatic potential maps were visualized via the Chemcraft program [89] and further quantified by the Multiwfn program [90].

## 3. Results

**Figure 1:**
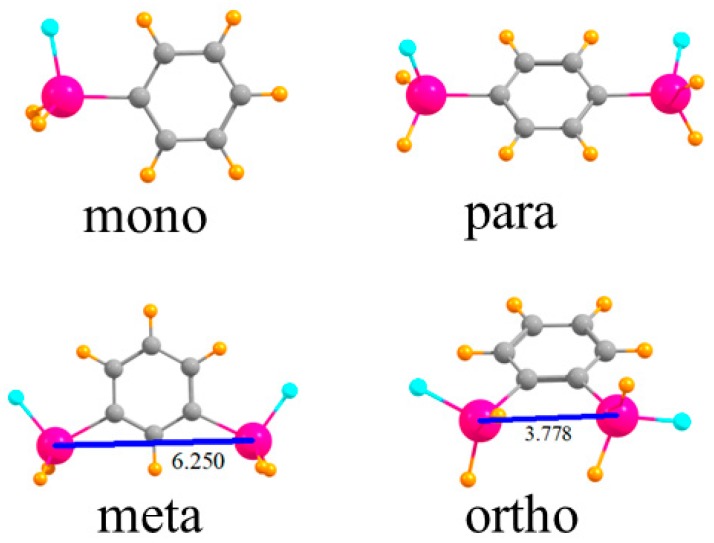
Geometries of mono- and disubstituted SnFH_2_ phenyl rings. Distances in Å.

The various monomers in which one or two SnFH_2_ groups are placed on a phenyl ring are displayed in Figure 1. First is the single SnFH_2_ group so as to permit the elucidation of the intrinsic nature of its binding properties to an anion. Adding a second such group, para to the first, permits the extraction of how this second group affects the anion binding to the first, but only indirectly, through the intermediacy of the intervening phenyl ring. When these two groups are placed closer together, in the meta position, there is the possibility they can both interact with the anion, but the 6.250 Å distance between the two Sn atoms would likely require some contraction for this to occur. The two Sn atoms are very much closer together, 3.778 Å, in the ortho configuration, and much more amenable to a bidentate interaction with an anion.

**Figure 2:**
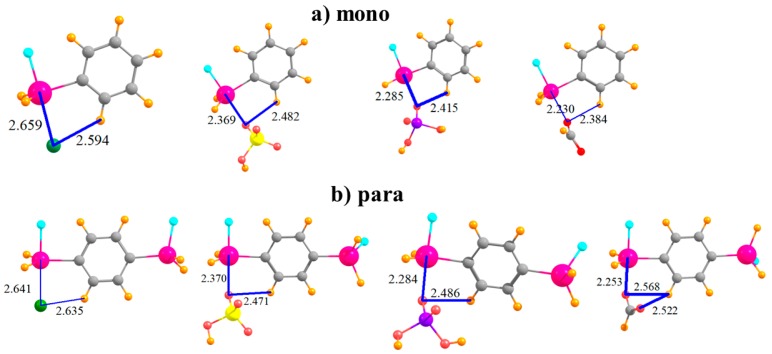
Complexes of Cl^−^, HSO_4_^−^, H_2_PO_4_^−^, and HCOO^−^ with (**a**) monosubstituted and (**b**) para disubstituted phenyl rings. Distances in Å.

The complexes formed by the mono-substituted molecule with each of the four anions are displayed in Figure 2a. The distance between the Sn atom and the Cl^−^ is somewhat larger than that to the nearest O atom of the other anions, likely due in large part to the larger size of the former atom. Also displayed in Figure 2 are distances between the anion and the proximate CH group of the phenyl ring since there is a possibility of some attraction in the form of a CH···O/Cl H-bond. The binding energy E_b_ of each complex is reported in the first row of Table 1. This quantity is followed by the interaction energy E_int_, which differs from E_b_ in that it refers to the monomers in their geometry within the complex, rather than as fully optimized monomers. (Both of these quantities have been corrected for basis set superposition errors which are reported in Table 2.) The binding energies lie in the range between 29 and 43 kcal/mol; E_int_ is somewhat larger, 37–55 kcal/mol. The ordering of the energetics of complexation diminishes in the order HCO_2_^−^ > Cl^−^ > H_2_PO_4_^−^ > HSO_4_^−^. The difference between E_b_ and E_int_ represents E_def_, the energy required to distort each monomer into the structure it adopts within the complex, listed in Table 3. It is important to note at the outset that even in the binding to the mono-substituted Lewis acids, there is a substantial deformation energy of more than 8 kcal/mol. Figure 2b shows that the addition of the second SnFH_2_ group para to the first has only a marginal effect on the intermolecular geometry. In terms of energy, there is a fairly uniform increment of 4–5 kcal/mol in E_b_, and a slightly larger increase in E_int_; the pattern of the four anions remains unchanged. The deformation energy rises, but again, only by a small amount.

**Figure 3:**
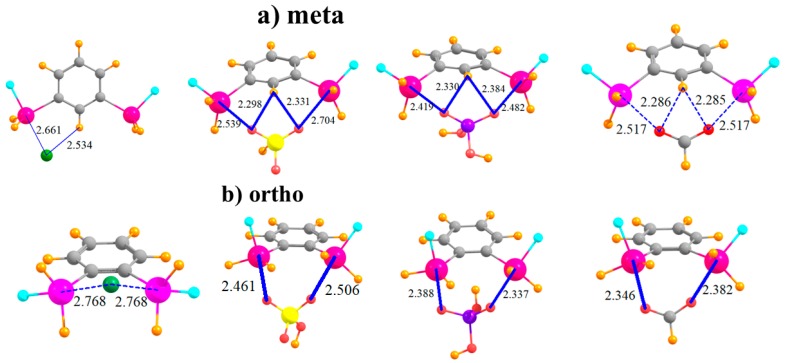
Complexes of Cl^−^, HSO_4_^−^, H_2_PO_4_^−^, and HCOO^−^ with (**a**) meta and (**b**) ortho disubstituted phenyl rings.

The placement of the second SnFH_2_ in the meta position raises the possibility of the anion interacting directly with two Sn atoms simultaneously, at least for the polyatomic anions. Indeed, the latter adopt nearly symmetric positions between the Sn atoms, with roughly equal pairs of R(Sn··O) distances, as seen in Figure 3a. Inspection of Table 4, which displays the shorter of the two distances in each case, shows that this bidentate structure comes at the expense of a stretched R(Sn··O) distance, longer by some 0.2 Å than in the para-substituted monodentate cases. As a result of the ability of the polyatomic anions to engage in two tetrel bonds, their binding energy of HSO_4_^−^ and H_2_PO_4_^−^ rises substantially relative to the para configuration, with less of a bump for HCO_2_^−^ and Cl^−^. Cl^−^ and HSO_4_^−^ reverse places for the meta structure, with the latter engaging in a stronger complex. In order to accommodate the anion, and to better engage in a pair of tetrel bonds, the Lewis acid molecule distorts so as to bring the two Sn atoms closer together. This intramolecular contraction is reported in Table 5, where it may be seen to be as much as 0.5 Å. (There is negligible change for Cl^−^ since this anion cannot form a bidentate complex with the meta molecule.) Despite this fairly large geometry change, the deformation energy is only slightly larger than in the para case, with an increment of only 2–4 kcal/mol. This small energy change underscores the flexibility of the meta system, which allows it to better bind with the anions. Finally, it might be noted that the meta complexes retain the fairly short H··X distances characteristic of a possible CH··X H-bond, which add to the stability of each complex.

The two Sn atoms are close enough together in the ortho configuration that all anions can engage in a bidentate pair of tetrel bonds, as displayed in Figure 3b. This ability raises the binding energy of the Cl^−^ by some 11 kcal/mol, and adds to the stability of the HCO_2_^−^ complex. The Cl^−^ and HSO_4_^−^ anions go back to their original energetic ordering, now that both engage in a pair of tetrel bonds. The transition from meta to ortho allows shorter R(Sn··O) bond distances. It is interesting to observe that unlike the meta structure where the two Sn atoms come closer together to interact with the anion, the reverse occurs for the ortho arrangement. That is, the Sn atoms move further apart in order to make room for the anion, with R(Sn···Sn) elongations of some 0.3–0.5 Å, as detailed in Table 5. As a result, the deformations of the meta and ortho systems are comparable. In any case, the anion lies well above the aromatic plane of the Lewis acid.

**Figure 4:**
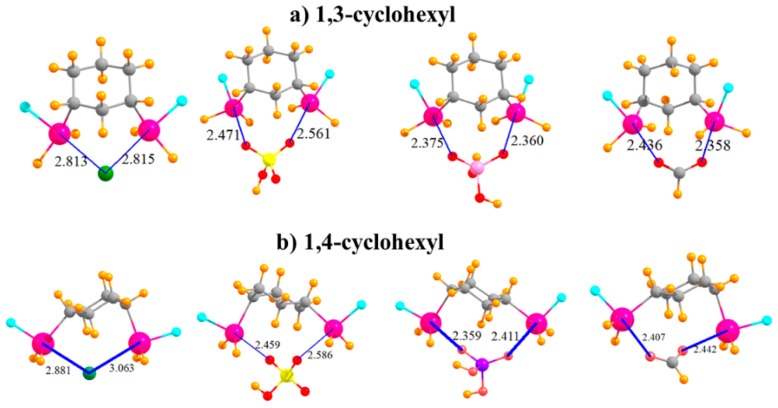
Complexes of Cl^−^, HSO_4_^−^, H_2_PO_4_^−^, and HCOO^−^ with (**a**) 1,3 and (**b**) 1,4 disubstituted cyclohexyl rings.

The question arises as to the effect of placing the two SnFH_2_ groups on an aromatic ring. This issue was investigated by removing the aromaticity and replacing phenyl with an aliphatic cyclohexyl ring. In order to mimic the meta substitution in the aromatic systems, the two SnFH_2_ groups were initially placed in 1,3 positions on the cyclohexyl ring. The geometries of the complexes with the anions are displayed in Figure 4a and show strong complexes with short R(Sn···X) distances. In the cases of HSO_4_^−^ and HCO_2_^−^, these distances are even shorter than in the meta aromatic systems. The switch from the meta aromatic to the 1,3 aliphatic Lewis acid has little effect on the binding energies of HSO_4_^−^ and H_2_PO_4_^−^, but enhances the binding of Cl^−^ and HCO_2_^−^. This loss of aromaticity also engenders a small increase in the deformation energy in order to accommodate the various anions. In connection with this distortion, unlike the meta aromatic case where the two Sn atoms move further apart in the complex, there is a very significant stretch of this interatomic distance in the aliphatic case. Moving the two SnFH_2_ groups further apart into 1,4 positions has relatively little effect on the properties of the complexes with the anions. Binding energies are stable to within a few kcal/mol, and the R(Sn···X) binding distances do not change much either. It is curious to note in Table 5 that whereas the two Sn atoms approach much closer to one another to accommodate the chloride, as well as HCO_2_^−^, they must stretch apart in the case of the H_2_PO_4_^−^. Regardless of stretch or contraction, the deformation energies of the 1,4 substituted cyclohexyl rings are not very different from their 1,3 congeners. Finally, it is worth noting that the 1,4 aliphatic ring can easily bind the anions in a bidentate fashion, while its para aromatic correlate is limited to only monodentate binding.

**Figure 5:**
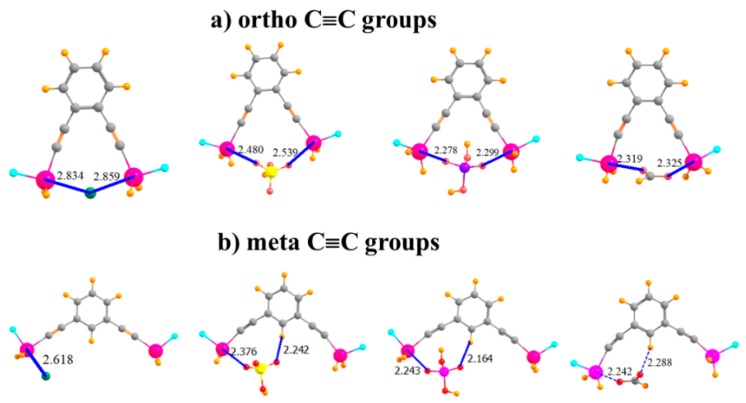
Of Cl^−^, HSO_4_^−^, H_2_PO_4_^−^, and HCOO^−^ with -C≡C- extensions on (**a**) ortho and (**b**) meta positions of phenyl ring.

Given the apparent flexibility of these binding units, one may wonder how far the Sn atoms can be placed apart so as to remove their ability to both bind the anion. The S···Sn distance was enlarged by adding a linear C≡C spacer unit separating each SnFH_2_ group from the aromatic ring, as depicted in Figure 5a. Indeed, this addition results in an R(Sn···Sn) distance in the monomer of 9.18 Å, compared to only 3.78 Å in the ortho-monomer of the aromatic system in Figure 3b. Remarkably, despite this long native distance, the larger molecule is fully capable of engaging in bidentate binding of the anions, as characterized in Figure 5a. Indeed, the binding energies are enhanced (with the exception of Cl^−^) by as much as 11 kcal/mol in the case of H_2_PO_4_^−^. Nor do the R(Sn···X) binding distances suffer at all from the C≡C spacers. One may say that the extra Sn···Sn distance allows the anions to fit in snugly between these atoms, without having to move out of the molecular plane, as was the case for the ortho-placement in Figure 3b, representing a better fit in some ways. The C≡C systems are also quite flexible, with the two Sn atoms able to move closer together by more than 1 Å to better bind to the anions, with little more deformation energy than for the other systems examined here.

By placing these C≡C groups in the meta positions, the Sn atoms are moved much further apart, to 10.84 Å in the monomer. As seen in Figure 5b, it is no longer possible for any of these anions to span both of the Sn atoms and engage in a bipodal complex. Consequently, the binding energies resemble those for the mono and para SnFH_2_ groups in the first and second rows of Table 1, along with similar R(Sn···X) distances. As in these earlier complexes, there is again the possibility of an auxiliary CH···O H-bond involving an aryl CH. Complexation with the anions pulls the two Sn atoms in closer together by various amounts ranging up to 1.5 Å, but the deformation energy remains comparatively small.

**Figure 6:**
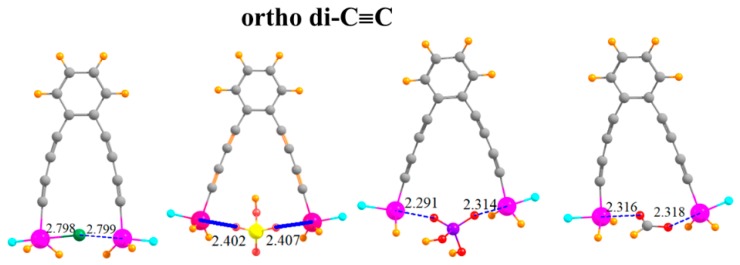
Complexes of Cl^−^, HSO_4_^−^, H_2_PO_4_^−^, and HCOO^−^ with -C≡C-C≡C- extensions on ortho positions of phenyl ring.

The arms on which the SnFH_2_ groups are situated can be lengthened by adding a second -C≡C- group to each arm, situated ortho to one another, as indicated in Figure 6. This pair of elongated arms increases the R(Sn···Sn) distance in the monomer to 9.18 Å. It is thus remarkable that there is sufficient flexibility in this molecule that the two SnFH_2_ groups can come close enough together to both grab onto the anion. The two Sn atoms are able to move together by as much as 4.5 Å (Table 5). There is a certain rise in the ensuing deformation energy, up to 21 kcal/mol, but the binding energies are quite high, equal to 65.7 kcal/mol for HCO_2_^−^. The interaction energies are even larger, as much as 86.7 kcal/mol. Despite the long separation between the Sn atoms in the monomer, the flexibility leads to little deterioration of the binding characteristics caused by the second C≡C group. Indeed, the binding is stronger in the latter systems by several measures.

**Figure 7:**
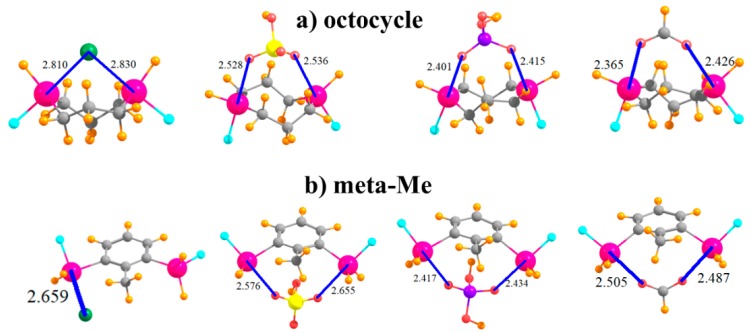
Complexes of Cl^−^, HSO_4_^−^, H_2_PO_4_^−^, and HCOO^−^ with SnFH_2_ groups (**a**) embedded in 8-membered ring, and (**b**) in 2,6 positions of toluene.

Another variation that was tested integrated the SnFH_2_ groups directly into an aliphatic ring, with this one containing eight atoms in an octocycle. With an inter-tin distance of 3.61 Å, the two Sn atoms are close enough together to both engage with the ions, as illustrated in Figure 7a. This complexation distorts the ring so as to bring the two tin atoms closer together by some 0.4–0.6 Å, with accompanying deformation energies not unlike the other systems, between 13 and 18 kcal/mol. This range is similar to, albeit slightly smaller than, the binding energies when the SnFH_2_ groups were appended to the cyclohexyl ring, in either 1,3 or 1,4 positions, and with comparable R(Sn···X) distances.

It was mentioned earlier that the CH group that lies in an ortho position to SnFH_2_ is capable of engaging in an H-bond with the anion. In order to test the magnitude of any such bond, this H atom was replaced by a methyl group in the context of the meta arrangement of SnFH_2_ groups, as displayed in Figure 7b. Comparison of the data in the last row of Table 1 with the meta row indicates that this H→Me mutation reduces the binding energy by some 3–6 kcal/mol. However, it also raises the deformation energy quite a bit, even though most of the geometric aspects of the binding, such as R(Sn···X), are not much affected by this substitution.

After proper attention is paid to vibrational energies and to entropic effects, the values of ∆G for the binding reactions are reported in Table 6. Given the reduction from two separate entities to a single complex as the reaction proceeds, it is not surprising to see that the values of ∆G are less negative than the electronic contributions to the binding energies in Table 1. This decrease lies in the range between 8 and 17 kcal/mol. The entropic contributions are somewhat smaller for Cl^−^ than for the other anions, averaging only 9 kcal/mol vs 15 kcal/mol for the others. As a result, the binding of Cl^−^ is diminished less than the larger anions, and so ∆G for Cl^−^ tends to be as negative as for the most strongly binding HCO_2_^−^ anion. The values of ∆G in Table 6 retain most of the patterns witnessed for ∆E above. The energetic ordering HSO_4_^−^ < HSO_4_^−^ < HCO_2_^−^ is continued for the most part, but with only a few exceptions, where ∆G places Cl^−^ on a par with HCO_2_^−^.

**Figure 8:**
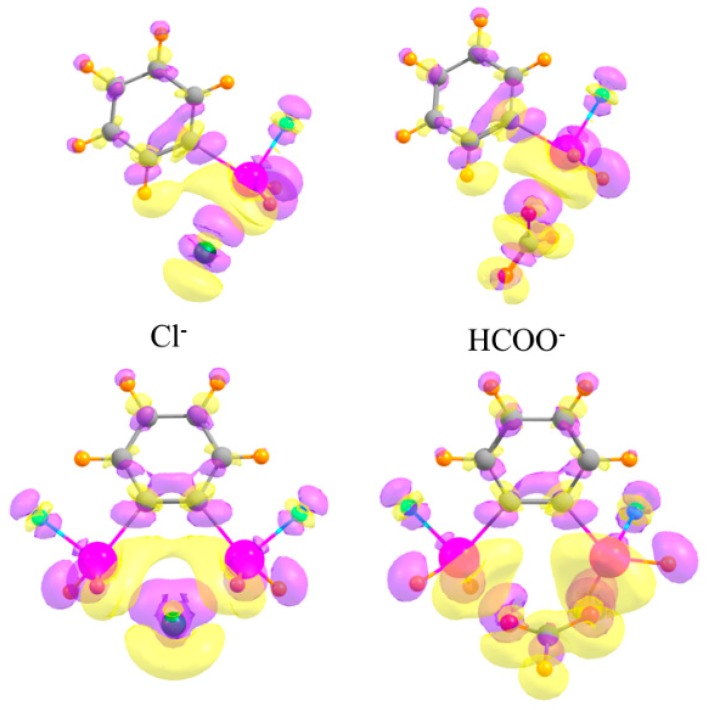
Density shifts caused by complexation. Upper diagrams refer to mono-SnFH_2_ substituted phenyl ring, lower to ortho-disubstituted. Cl^−^ anion on the left and HCOO^−^ on the right. Purple and yellow regions correspond to accumulation and depletion of density, respectively, with contour shown Δρ = ±0.002 au.

As a final consideration, the changes undergone by the electron densities of the two monomers as they coalesce into a dimer are typically characteristic of each type of interaction. The top half of Figure 8 depicts the electron density shifts when the mono-SnFH_2_ substituted phenyl ring is combined with the monatomic Cl^−^ anion (on the left) and a polyatomic HCOO^−^ anion (on the right). One sees in each case a pattern along the X···Sn bond axis, wherein a purple increase is observed near the Cl/O atom, and a yellow density loss near the Sn. This fingerprint is typical of tetrel bonds, as well as their pnicogen, chalcogen, and halogen cousins, as well as the more commonly studied H-bond [40,91,92,93,94,95,96,97]. This pattern also occurs for the disubstituted phenyl receptors in the lower half of Figure 8, and reinforces the idea that the anion engages in a pair of tetrel bonds, one with each Sn atom.

## 4. Related Properties of Monomers

It is interesting to conjecture as to the order found here for the binding of the four anions: HSO_4_^−^ < HSO_4_^−^ < HCO_2_^−^, with Cl^−^ generally intermediate between HSO_4_^−^ and HCO_2_^−^. One consideration might be the partial negative charge on the O/Cl atom that interacts with the Sn atoms. Within the Mulliken framework, these charges are less than -1, as displayed in the first row of Table 7. However, the O charge for HCO_2_^−^ is less negative than for the various other anions, in contrast to its stronger binding. The same pattern is noted for the natural charges in the next row of the table. More consistent with the energetics is the value of V_s,min_, the minimum of the electrostatic potential on the pertinent atomic center, for which the formate O atom has the most negative value, followed by H_2_PO_4_^−^ and then HSO_4_^−^. (As a spherical unit, Cl^−^ would have no minimum on its surface.)

In connection with the various Lewis acids, it is common in the literature to observe a correlation between the binding energetics with a Lewis base and the value of V_s,max_, which lies along the approximate line connecting the interacting atoms. The values of V_s,max_ are listed for each of the Lewis acids in Table 8, all of which lie opposite the Sn-F covalent bond. There is one anomaly to note, which is the absence of any maximum in the electrostatic potential of the octocyclic system directly opposite the F atom of either SnFH_2_ group, which may be a reflection of the close proximity of the two Sn atoms in the monomer.

**Figure 9:**
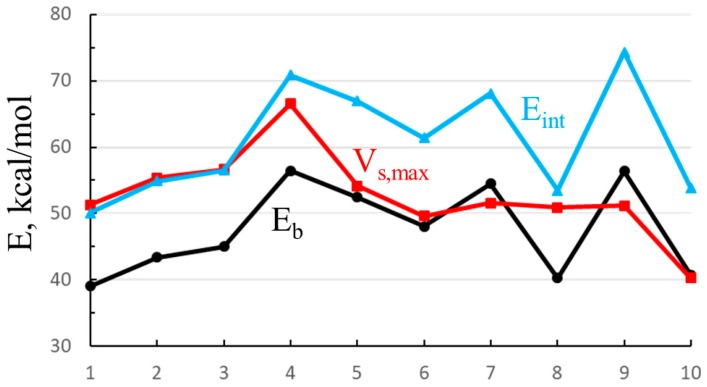
Binding (E_b_) and interaction (E_int_) energies of complexes of Cl^−^ with each of the monomers (numbering in Table 8), along with maximum in electrostatic potential opposite the F atom of SnFH_2_ group.

One can see some resemblance between V_s,max_ and the energetics in Table 1. In order to more closely probe any similarities, V_s,max_ is plotted as the red curve in Figure 9, along with both the binding (black) and interaction (blue) energies of the Cl^−^ anion for illustrative purposes. The numbers along the horizontal axis refer to those in Table 8. First, the interaction and binding energies are very nearly parallel to one another, displaced by the deformation energy, which is roughly comparable from one complex to the next. V_s,max_ exhibits some of the same trends: It climbs in the mono < para < meta < ortho order (points 1–4), followed by the dropoff down to point 6. However, V_s,max_ remains relatively constant between 6 and 9, showing none of the zig-zag behavior of the energetics as the C≡C groups are extending the arms which house the SnFH_2_ groups. In summary, the energetics are only weakly correlated with the σ-hole. The correlation coefficient between this quantity and E_b_ (for the illustrative Cl^−^) is only 0.48; that with E_int_ is 0.40.

## 5. Discussion

A primary goal had been the design of a receptor that shows a strong preference for a polyatomic anion whose O atoms are some distance apart, as compared to a simple halide. The guiding idea had been that only such a polyatomic would be able to span a long distance between the two Sn atoms built into the receptor so as to engage in a pair of Sn···O tetrel bonds. However, this attempt has not yielded the desired results. As a starting point, the single SnFH_2_ group placed on a phenyl ring forms a tetrel bond with monoatomic Cl^−^ that is stronger than both HSO_4_^−^ and H_2_PO_4_^−^, surpassed by HCO_2_^−^. When a second SnFH_2_ group is placed on the phenyl ring, meta to the first, one sees the desired geometry in the complexes; viz. the polyatomics can span the two Sn atoms, while Cl^−^ cannot. As such, the polyatomics enjoy a bit of a boost over the Cl^−^, but not enough to raise the binding energy of HSO_4_^−^ over that of Cl^−^.

Another strategy placed the two tetrels on an aliphatic cyclohexyl ring. However, regardless of whether the two groups are in 1,3 or a 1,4 positions relative to one another, the flexibility of the ring is such that all anions, including the monatomic Cl^−^, are able to span the Sn···Sn distance and engage in a pair of tetrel bonds, offering no energetic advantage to the polyatomics.

Returning to the original aromatic ring, the two SnFH_2_ groups were placed at the end of -C≡C- arms, adding to their separation. When these two arms lie ortho to one another, there is sufficient flexibility that all anions can engage in tetrel bonds to both Sn atoms. Further separating the Sn atoms by moving the two -C≡C- arms to meta positions removes the possibility of any of the anions, polyatomic as well as monatomic, from spanning the distance between them. The Sn···Sn separation can also be increased by lengthening each arm to a -C≡C-C≡C- unit. Even though this distance is more than 9 Å in the uncomplexed ortho monomer, the arms have sufficient flexibility that the two Sn atoms on their ends move in toward the anion, allowing even the Cl^−^ to engage in a pair of tetrel bonds. The net result of these arms, ortho or meta, short or long, on the various binding energies is thus a small one, merely switching the relative stabilities of Cl^−^ and H_2_PO_4_^−^.

Instead of employing the SnFH_2_ groups as appendages on the receptors, they were incorporated directly into the ring itself, in an octocycle. Placing them in 1,4 positions, on opposite ends of the aliphatic ring, still left the Sn atoms only 3.6 Å apart, able to engage in a pair of tetrel bonds with each of the anions, and offering no advantage to any of them.

The tetrel bonds here are charge-assisted in the sense that an anion is involved in each. Additionally, there are two such bonds in most of these complexes. Consequently, the interaction energies are quite large, ranging up to as much as 87 kcal/mol. They are thus a powerful force, rearranging the receptor to better engage in these bonds. The anions pull and tug on the Sn atoms, pulling them closer together or further apart, so as to better accommodate the incipient tetrel bonds. These inter-tin distance changes can be as much as 4.5 Å in one case. It is thus not surprising to note the fairly large deformation energies that accompany these geometrical changes, in the 8–25 kcal/mol range, that detract from the final binding energies.

The particular halide chosen for intensive study here has been chloride. The use of larger halides would normally be expected to bind less intensely with the receptor, due, in large part, to their larger size and more diffuse charge. Indeed, calculations of Br^−^ and I^−^ with the ortho receptor in Figure 1 do lead to diminished binding, with E_b_ equal to 48.8 and 39.7 kcal/mol, respectively, in comparison to 56.5 kcal/mol for Cl^−^. Their larger size would also lead to less sensitivity to longer Sn···Sn distances, as they could more easily span the two Sn atoms. Previous calculations [71,72,76] suggest that F^−^ would engage in very strong interactions with a single Sn atom, bordering on a true covalent bond.

While the intensity of the σ-hole on each Sn atom is clearly an important factor in the strength of the binding, it does not play a dominant role. The trends of V_s,max_ from one Lewis acid to the next only roughly parallel the binding energies. The energies required to deform the receptor in order to accommodate each anion are large and certainly play an important part. Another factor is the ability of the anion to bridge the two Sn atoms, which prevents a perfect alignment with the σ-hole of either Sn atom individually. There is also the possibility that the negative ions can engage in attractive interactions with atoms other than the Sn atoms. For example, there is evidence of aryl CH···O H-bonds in certain configurations.

In conclusion, various neutral Lewis acid Sn-receptors are capable of engaging in strong interactions with Cl^−^, as well as several polyatomic O-containing monoanions. There is a general pattern that HCO_2_^−^ engages in the strongest such interaction, followed by Cl^−^, and then by H_2_PO_4_^−^ and HSO_4_^−^. The receptors show surprising flexibility in order to bind the anion in a bipodal fashion, with a pair of Sn···X tetrel bonds of comparable strength. The geometrical distortions undergone by the receptor lead to fairly large internal deformation energies, the cost of which is repaid by the strong binding to the anion. Even though the polyatomic anions are better able to span the distance between the two Sn atoms, forming two Sn···O tetrel bonds, each to a different O atom, this ability does not offer a dramatic benefit over the simpler monatomic Cl^−^ anion.

## Figures and Tables

**Table 1:** Binding (E_b_) and interaction (E_int_) energies (kcal/mol) for pairing of indicated Lewis acids with anions.

	E_b_				E_int_			
	Cl^−^	HSO_4_^−^	H_2_PO_4_^−^	HCO_2_^−^	Cl^−^	HSO_4_^−^	H_2_PO_4_^−^	HCO_2_^−^
mono	39.06	29.12	35.43	43.24	50.10	37.32	46.17	54.91
para	43.37	33.49	39.26	48.74	54.92	42.26	51.94	61.16
meta	45.03	39.37	50.87	51.18	56.53	50.35	67.35	65.29
ortho	56.45	39.12	53.18	59.00	70.88	49.95	72.87	74.35
1,3-cyclohex	52.47	38.98	50.83	57.23	66.96	50.98	71.01	72.81
1,4-cyclohex	48.09	41.32	52.76	56.32	61.42	53.25	68.69	72.12
ortho C≡C	54.46	45.37	64.01	66.38	68.11	57.19	86.13	82.35
meta C≡C	40.28	31.99	42.48	45.76	53.46	41.15	57.70	60.66
ortho di-C≡C	56.41	51.58	58.65	65.74	74.34	65.92	78.29	86.65
octocycle	48.14	37.58	46.93	54.91	63.76	50.72	64.55	71.71
meta-Me	40.72	34.17	48.19	45.19	53.88	50.56	73.51	65.85

**Table 2:** Basis set superposition error (kcal/mol) as corrected by counterpoise procedure.

	E_BSSE_			
	Cl^−^	HSO_4_^−^	H_2_PO_4_^−^	HCO_2_^−^
mono	0.41	0.41	1.58	0.95
para	0.42	1.67	1.83	1.03
meta	0.39	1.90	2.37	1.26
ortho	0.58	1.73	2.59	1.36
1,3-cyclohex	0.65	2.09	2.44	1.43
1,4-cyclohex	0.62	2.22	2.79	1.54
ortho C≡C	0.45	1.80	2.35	1.47
meta C≡C	0.38	1.48	1.84	0.97
ortho di-C≡C	0.46	1.82	1.98	1.45
octocycle	0.83	2.56	3.09	1.84
meta-Me	0.50	2.38	3.18	1.49

**Table 3:** Deformation energy (kcal/mol) required for optimized monomers to adapt to geometry within each complex.

	E_def_			
	Cl^−^	HSO_4_^−^	H_2_PO_4_^−^	HCO_2_^−^
mono	11.04	8.20	10.74	11.67
para	11.55	8.77	12.68	12.42
meta	11.50	10.98	16.48	14.11
ortho	14.43	10.83	19.69	15.35
1,3-cyclohex	14.49	12.00	20.18	15.58
1,4-cyclohex	13.33	11.93	15.93	15.80
ortho C≡C	13.65	11.82	22.12	15.97
meta C≡C	13.18	9.16	15.22	14.90
ortho di-C≡C	17.93	14.34	19.64	20.91
octocycle	15.62	13.14	17.62	16.80
meta-Me	13.16	16.39	25.32	20.66

**Table 4:** Shorter of two R(Sn···X) (X = Cl,O) distances (Å) in indicated complexes.

	R, Å			
	Cl^−^	HSO_4_^−^	H_2_PO_4_^−^	HCO_2_^−^
mono	2.594	2.369	2.285	2.230
para	2.641	2.370	2.284	2.253
meta	2.661	2.539	2.419	2.517
ortho	2.768	2.461	2.354	2.346
1,3-cyclohex	2.813	2.360	2.471	2.358
1,4-cyclohex	2.881	2.459	2.359	2.407
ortho C≡C	2.834	2.480	2.278	2.319
meta C≡C	2.618	2.376	2.243	2.242
ortho di-C≡C	2.798	2.402	2.291	2.316
octocycle	2.810	2.528	2.401	2.365
meta-Me	2.659	2.576	2.417	2.487

**Table 5:** R(Sn···Sn) distance (Å) in Lewis acid and its change caused by complexation with anion.

	R (monomer)	ΔR			
		Cl^−^	HSO_4_^−^	H_2_PO_4_^−^	HCO_2_^−^
meta	6.250	−0.052	−0.348	−0.382	−0.503
ortho	3.778	0.284	0.215	0.318	0.252
1,3-cyclohex	3.768	0.277	0.352	0.501	0.327
1,4-cyclohex	5.575	−0.500	−0.037	0.513	−0.221
ortho C≡C	6.501	−1.144	−0.069	−0.018	−0.861
meta C≡C	10.841	−0.383	−1.226	−0.717	−1.459
ortho di-C≡C	9.181	−4.499	−2.349	−2.701	−3.489
octocycle	3.613	0.400	0.640	0.621	0.560
meta-Me	6.258	0.017	−0.324	−0.367	−0.497

**Table 6:** Binding free energies (kcal/mol) at 25 C for pairing of indicated Lewis acids with anions.

	−∆G, kcal/mol			
	Cl^−^	HSO_4_^−^	H_2_PO_4_^−^	HCO_2_^−^
mono	31.20	16.58	22.99	30.49
para	35.04	21.06	25.30	35.51
meta	36.22	24.52	35.44	36.00
ortho	46.64	25.41	37.78	43.83
1,3-cyclohex	43.04	24.32	35.75	42.02
1,4-cyclohex	36.97	24.10	35.78	39.66
ortho C≡C	44.08	30.10	47.09	50.09
meta C≡C	31.23	18.48	27.79	31.08
di-C≡C	46.58	35.50	42.51	51.47
octo	39.78	24.34	32.40	40.53
meta-Me	31.76	18.65	31.76	30.07

**Table 7:** Measures of negative charge on O/Cl atom interacting with Sn.

	Cl^−^	HSO_4_^−^	H_2_PO_4_^−^	HCO_2_^−^
Mulliken charge, e	−1.00	−0.85	−0.85	−0.65
natural charge, e	−1.00	−1.03	−1.20	−0.84
V_s,min_^a^, kcal/mol	−	−130	−135	−155

^a^ evaluated on the ρ = 0.001 au isodensity surface.

**Table 8:** Maximum of electrostatic potential opposite F atom of SnFH_2_ group.

		V_s,max_, kcal/mol
1	mono	51.32
2	para	55.40
3	meta	56.63
4	ortho	66.54
5	1,3-cyclohex	54.18
6	1,4-cyclohex	49.62
7	ortho C≡C	51.59
8	meta C≡C	50.92
9	ortho di-C≡C	51.18
-	octo	^a^
10	meta-Me	40.30

^a^ no max in MEP opposite F atoms.
